# Diagnostic Imaging and Clinical Implications of Heterotopic Ossification After Total Ankle Arthroplasty: A Systematic Review for Surgical Strategy

**DOI:** 10.3390/diagnostics15172203

**Published:** 2025-08-29

**Authors:** Simone Ottavio Zielli, Francesca Veronesi, Giulia Sacchi, Antonio Mazzotti, Cesare Faldini, Gianluca Giavaresi

**Affiliations:** 1 1st Orthopaedic and Traumatologic Clinic, IRCCS Istituto Ortopedico Rizzoli, Via Putti 1, 40136 Bologna, Italy; simoneottavio.zielli@ior.it (S.O.Z.); antonio.mazzotti@ior.it (A.M.); cesare.faldini@ior.it (C.F.); 2 Surgical Sciences and Technologies, IRCCS Istituto Ortopedico Rizzoli, Via Di Barbiano 1/10, 40136 Bologna, Italy; giulia.sacchi@ior.it (G.S.); gianluca.giavaresi@ior.it (G.G.); 3 Department of Biomedical and Neuromotor Sciences (DIBINEM), Alma Mater Studiorum University of Bologna, 40126 Bologna, Italy

**Keywords:** total ankle arthroplasty, aging, heterotopic ossification, systematic review, complications

## Abstract

**Background**: Heterotopic ossification (HO) is a frequent radiographic finding after total ankle arthroplasty (TAA), but its clinical relevance, diagnostic criteria, and prognostic implications remain uncertain. This systematic review summarizes current evidence on HO incidence, distribution, severity, risk factors, clinical impact, and diagnostic/prognostic value to inform surgical decision-making regarding approach, implant design, and revision strategies. **Methods**: A systematic review was conducted according to PRISMA guidelines using PubMed, Web of Science, and Scopus databases and the following search string “heterotopic ossification” AND “ankle” (February 2015–February 2025). Twenty-two studies were included, most of which were retrospective and varied in methodological quality. Data were extracted on HO incidence, severity, clinical relevance, and factors associated with diagnosis and management. **Results**: HO incidence varied widely across studies. No significant associations were found between HO and surgical variables such as approach (all studies used the anterior approach) or coronal alignment. HO presence did not consistently correlate with reduced postoperative range of motion and radiographic follow-up duration. Implant design appeared to influence anatomical distribution in some comparative studies, though without statistical significance. Reoperations specifically for HO excision were rare and mainly performed for mechanical complications (impingement or osteolysis) rather than HO severity itself. **Conclusions**: Although HO is a frequent finding after TAA, its clinical impact appears limited and largely unpredictable. Diagnostic tools are currently limited to conventional radiography, and no reliable prognostic markers exist. Further high-quality studies are needed to define standardized diagnostic criteria and clarify the prognostic role of HO in long-term outcomes.

## 1. Introduction

Total ankle arthroplasty (TAA) has become an established surgical option for patients with end-stage ankle arthritis [1,2,3], aiming to relieve pain, preserve mobility, and restore a more physiological gait [3,4]. Continuous improvements in implant design, biomaterials, and surgical techniques over the past decades have significantly increased the procedure’s popularity and improved mid- to long-term survivorship, with modern implants reporting five-year survival rates of 92–97% [5,6,7]. Despite these advancements, complications remain a concern, particularly those affecting long-term outcomes and revision rates. Intraoperative risks include fractures and iatrogenic neurovascular injuries, while postoperative issues may involve wound healing problems and infections [8].

Among the complications reported after TAA, heterotopic ossification (HO) is a frequent radiographic finding [9]. HO is defined as the abnormal formation of bone within soft tissues, often triggered by local inflammation and osteoinductive factors. Various mechanisms have been proposed to explain this process, including the role of microbial bioburden in creating a local osteogenic environment that stimulates resident progenitor cells to form ectopic bone [10,11].

While extensively studied in other joints such as the hip and elbow, where incidence rates may reach 90% after arthroplasty [12], its clinical relevance following TAA is still debated and is presumed to result from a complex interplay of systemic and local factors [11,13,14]. HO may potentially cause pain, mechanical impingement, and limited range of motion (ROM), but many cases remain asymptomatic, and its true prognostic value is unclear [15].

Several surgical and patient-related risk factors for HO formation have been described, including extensive soft tissue dissection, bone debris, malalignment, prolonged operative times, postoperative hematoma, male sex, older age, and a history of heterotopic ossification in other joints [16,17]. Additionally, implant design and positioning may influence the anatomical distribution and severity of HO, although current evidence is heterogeneous and inconclusive.

Despite the frequency of HO reported in the literature, standardized diagnostic criteria and consistent reporting are lacking. Radiographic classifications, such as the Brooker system modified for the ankle, are inconsistently applied, and no validated biochemical or imaging biomarkers are available to predict clinically significant HO [18,19]. Furthermore, the relationship between HO severity, ROM, pain, and revision surgery remains uncertain, leaving clinicians without robust guidelines to prevent or manage this condition. Various biochemical markers of bone metabolism have been explored for HO prediction, though none have demonstrated reliable diagnostic or prognostic value. Currently, the most effective prophylactic strategies remain limited to radiotherapy and nonsteroidal anti-inflammatory drugs (NSAIDs) [12].

Surgical variables, implant design, and follow-up duration on HO development, and to provide evidence-based recommendations for surgical strategy and patient management.

Despite increasing awareness of its clinical relevance, evidence-based guidelines are still lacking regarding how modifiable surgical factors—such as implant selection, surgical approach, and prosthetic alignment—may influence HO incidence and severity. This gap hinders the development of intraoperative strategies tailored to minimize HO formation and improve patient outcomes.

This systematic review aims to synthesize current evidence on the incidence, anatomical distribution, radiological severity, risk factors, clinical outcomes, and diagnostic/prognostic value of HO after TAA. By analyzing studies published over the past decade, this review seeks to clarify whether HO development is influenced by intraoperative variables such as surgical approach and coronal alignment, and whether it is associated with a reduction in postoperative ROM, whether implant design affects the anatomical distribution of HO, whether HO incidence correlates with radiographic follow-up duration, potentially leading to underreporting in short-term studies, and, finally, the contribution of HO to TAA reoperations, and whether specific HO grades or distribution patterns are more likely to necessitate surgical revision.

## 2. Materials and Methods

This systematic review was conducted in accordance with the PICOS (Population, Intervention, Comparison, Outcomes, Study design) framework. Studies were considered eligible if they met the following criteria: included patients with end stage ankle arthritis (Population), treated with TAA (Intervention), featured comparisons either pre- versus post-surgery or between different prosthesis designs (Comparison), reported the presence of HO (Outcomes), and were clinical studies of any design (Study Design) (Figure S1). This systematic review has been registered on PROSPERO (ID CRD420250655560).

### 2.1. Data Sources and Search Strategy

A comprehensive literature search was conducted on 28 February 2025, using the PubMed, Web of Science, and Scopus databases. The following search string was applied across all databases: (heterotopic ossification) AND (ankle).

Filters included English language and a publication date range from 28 February 2015 to 28 February 2025.

**Figure 1:**
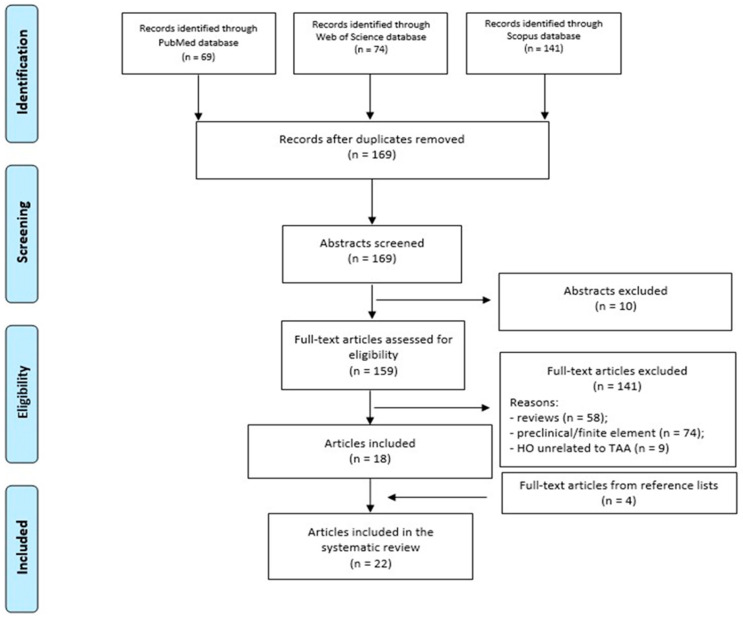
PRISMA flowchart of the study selection process.

The review adhered to the Preferred Reporting Items for Systematic Reviews and Meta-Analyses (PRISMA) guidelines. The study selection process is summarized in the PRISMA flowchart (Figure 1).

### 2.2. Study Selection

Duplicate records were removed using EndNote 21. The remaining articles were screened by two authors (FV and SOZ) based on title and abstract, according to the following inclusion criteria: clinical studies of any level of evidence involving TAA performed for ankle injuries, with reported postoperative HO. Exclusion criteria were: preclinical studies, reviews, book chapters, technical notes, comments, or any study that did not report HO among complications.

In cases where the abstract did not provide sufficient information, the full text was retrieved and assessed against the inclusion/exclusion criteria. The study selection process was independently performed by two reviewers (FV and SOZ), with disagreements resolved by a third author (GG).

Reference lists of all included studies were also reviewed to identify additional eligible publications.

### 2.3. Data Extraction

Two authors (FV and SOZ) independently extracted data using a standardized form. The following information was collected (Table 1):

- Reference (Ref.), study type, and patient treatment period (yrs);
- Number of patients (pts) and joints treated, including patient sex and age;
- Indications for surgery and their respective percentages;
- Type of prosthetic implant and mean follow-up duration in months (mo);
- Incidence of complications, HO rate, HO localization, and HO severity.

### 2.4. Risk of Bias Assessment

The risk of bias in each included study was independently evaluated by two reviewers (FV and SOZ), with disagreements resolved through consensus with a third author (GG).

The following two validated tools were used:

1. Downs and Black checklist [20], consisting of 26 items across five domains:
  - Reporting (9 items);
  - External validity (3 items);
  - Bias (7 items);
  - Confounding (6 items);
  - Power (1 item);
  - The maximum score is 31 points.
2. Modified Coleman Methodology Score (mCMS) [21], comprising 11 criteria:
  - Study size;
  - Mean follow-up duration;
  - Number of surgical procedures per outcome;
  - Study type;
  - Surgical technique description;
  - Postoperative rehabilitation details;
  - Use of MRI outcomes;
  - Inclusion of histological outcomes;
  - Outcome measures;
  - Clinical outcome assessment methods;
  - Description of subject selection process.

### 2.5. Statistical Analysis

Descriptive statistics were used to summarize study characteristics, including implant type, surgical approach, HO incidence and distribution, and reoperation rates.

Associations between HO and various clinical or radiographic parameters were evaluated using linear and multiple linear regression analyses:

1. Simple linear regression was applied to assess:
  - The relationship between HO incidence and change in ROM, using pre- and post-operative ROM data from four studies.
  - The association between HO incidence and radiographic follow-up duration. The Durbin–Watson test was performed to check for autocorrelation in residuals.
2. Multiple linear regression models were developed to:
  - Analyze whether the distribution of Brooker grades (I–III) predicted the likelihood of HO resection. Grade IV was excluded due to insufficient data.
  - Explore whether overall reoperation rates were influenced by a combination of factors, including HO incidence, HO resection rate, infection, implant subsidence, and loosening.

All statistical analyses were conducted using Jamovi version 2.6.26. A *p*-value of <0.05 was considered statistically significant.

## 3. Results

A total of 284 records were identified through database searches (PubMed: *n* = 69; Web of Science: *n* = 74; Scopus: *n* = 141). After duplicate removal (*n* = 115), 169 unique studies remained for screening. Following title and abstract evaluation, 10 studies were excluded due to irrelevance, and 141 were removed for being reviews (*n* = 58), preclinical/finite element studies (*n* = 74), or for focusing on ossifications unrelated to TAA, particularly in soft tissues (*n* = 9). Eighteen studies met the eligibility criteria, and an additional four were identified via reference screening, resulting in 22 studies included in the final analysis [7,12,22,23,24,25,26,27,28,29,30,31,32,33,34,35,36,37,38,39,40,41] (Figure 1) (Table 1).

**Table 1:** Summary of included study results.

Ref.	Type of Study (yrs)	Pts/N° of Joints (F/M and Age)	Indications for Surgery (%)	Type of Implant (%) Mean F-Up (mo)	Complications (%)	HO (%) Localization, Severity
Deleu, 2015 [22]	Retrospective case series (2008–2012)	50/50 (25/25; 55 ± 12 yrs)	Post-traumatic OA (68%); Inflammatory OA (18%); Primary OA (14%)	HINTEGRA (100%). 45 mo	Osteolysis (48%); Additional surgery (18%); Lucency (14%); Revision (10%)	Total: 48%. Posterior: 100%. Grade I (42%), Grade II (29%), Grade III (17%), Grade IV (12%)
Manegold, 2017 [12]	Retrospective case series (2005–2009)	84/88 (38/46; 55 ± 14 yrs)	Post-traumatic OA (70%); Primary OA (22%); Secondary OA (8%)	HINTEGRA (100%). 36 mo	n.r.	Total: 99%. Anterior (26%), posterior (100%), medial (23%), lateral (55%)
Clifton, 2021 [23]	Retrospective case serie (2010–2014)	70/70 (16/54; mean 69 yrs)	Primary OA (64%); Post-traumatic OA (21%); Unknown (10%) Inflammatory OA (3%); Others, AVN (2%)	HINTEGRA (100%). 77 mo	Cysts (40%); Revision (16%); Re-operation (13%); Minor (13%); Major (4%); Intermediate (3%); i.o. fracture (1%)	Total: 1%
Lee, 2018 [7]	Prospective case series (2005–2012)	144/144 (58/86; Mean 61 yrs)	Post-traumatic OA (59%); Primary OA (41%)	HINTEGRA (100%). 88 mo	Major (18%); Revision (17%); Minor (7%)	Total: 7%
Lee, 2019 [24]	Prospective case series (2005–2015)	123/123 (54/69; Mean 56 yrs)	Post-traumtaic OA (64%); Primary OA (30%); RA (6%)	HINTEGRA (100%). 78 mo	Major (28%); Revision (12%); Minor (11%)	Total: 8%.
Haytmanek, 2015 [25]	Retrospective case series (1998–2007)	76/79 (n.r.; 62 ± 11 yrs)	Post-traumatic OA (53%); Primary OA (33%); RA (13%); Hemochromatosis (1%)	STAR (100%). 96 mo	Lucency (56%); Revision (28%); Required secondary procedures (4%)	Total: 100%. Grade I (14%), Grade II (32%), Grande III (52%), Grade IV (2%)
Jastifer, 2015 [26]	Retrospective case series (1998–2003)	18/18 (8/10; Mean 61 yrs)	Post-traumatic OA (77%); Primary OA (17%); RA (6%)	STAR (100%). 151 mo	Swelling (55%); Revision (39%); Talar subsidence 2–5 mm (11%); Talar subsidence > 5 mm (6%)	Total: 78%.
Palanca, 2018 [27]	Retrospective case series (1998–2000)	24/24 (n.r.; Mean 74 yrs)	n.r.	STAR (100%). 188 mo	Cysts (14%); Osteolysis (14%); Revision (13%); Talar subsidence < 5 mm (10%); Talar subsidence > 5 mm (5%)	Total: 54%. Medial malleolus (54%), posterior tibia (69%). Grade III (23%)
Kerkhoff, 2016 [28]	Prospective case series (1999–2008)	134/134 (84/50; 59 ± 13 yrs)	RA (43%); Post-traumatic OA (33%); Primary OA (25%); Hemochromatosis (1%)	STAR (100%). 90 mo	Cysts (60%); Nonrevision secondary procedures (16%); Prosthesis failure (15%); Revision (10%); Lucency (9%); Re-operation (5%)	Total: 98%. Grade III (>50%)
Jamjoom, 2022 [29]	Retrospective case series (2016–2019)	28/29 (10/18; mean 68 yrs)	Revision TAA due to: Aseptic loosening of talar and tibial components (84%); Insert wear (10%); Talar malalignment (3%)	INBONE II (100%). 40 mo	Subsidence (31%); Loosening (21%); Osteolysis (17%); Re-operation (7%); i.o. deep peroneal nerve injury (3%)	Total: 31%. Tibia (45%), Talus (7%), Both (10%). Grade I (44%), Grade II (12%), Grade III (44%)
Rushing, 2021 [30]	Retrospective case series (2010–2014)	15/15 (8/7; Mean 63 yrs)	n.r.	INBONE II (100%). 85 mo	Re-operation (33%); Lucency (27%); Non–implant-related revision (27%); Minor (20%); Major (7%); Intermediate (7%)	Total: 67%. Anterior (7%), Posterior (40%), Anterior and posterior 20%. Grade I (30%), Grade II (20%), Grade III (30%), Grade IV (20%)
Bianchi, 2021 [31]	Retrospective case series (2004–2009)	34/34 (17/17; 54 ± 12 yrs)	Post-traumatic OA (73%); Primary OA (21%); RA (6%)	BOX (100%). 143 mo	Lucency (94%); Revision (59%); Re-operation (37%); Cysts (6%)	Total: 91%. Grade III-IV
Wan, 2018 [32]	Retrospective case series (2012–2015)	59/59 (28/31; Mean 64 yrs)	Post-traumatic OA (85%); Degenerative OA (8%); RA (5%); Tuberculous arthritis (2%)	Salto mobile-bearing. 36 mo	Osteolysis (46%); Re-operation (12%); Revision (5%); Progressive subtalar arthritis (2%)	Total: 22%. Grade I (62%), Grade II (38%)
Van Es, 2022 [33]	Retrospective case series (2004–2012)	237/254 (117/120; 59 ± 11 yrs)	Post-traumatic OA (59%); RA (25%); Idiopathic OA (9%); Hemochromatosis (2%); AVN (2%); Juvenile chronic arthritis (1%); Arthritic psoriasis (<1%); Ankylosing arthritis (<1%); Postinfectious OA (<1%); Ehlers-Danlos syndrome (<1%)	CCI evolution implant (100%). 83 mo	Re-operation (53%); Revision (22%)	Total: 13%
Penner, 2018 [34]	Prospective, case series (2013–2015)	67/67 (30/37; Mean 62 yrs)	Post-traumatic OA (70%); Inflammatory OA (6%); Primary OA (16%); Secondary OA (8%)	INFINITY (100%). 35 mo	Re-operation (9%); Revision (3%)	Total: 3%
Rushing, 2021 [35]	Prospective case series (2016–2018)	32/32 (14/18; Mean 61 yrs)	n.r.	CADENCE (100%). 24 mo	Lucency (41%); Minor (22%); Re-operation (19%); Major (13%); Intermediate (6%); Revision (6%)	Total: 31% All posterior
**COMPARATIVE**						
Jung, 2015 [36]	Retrospective comparative case series (2004–2012)	52/54 (24/28; Mean 65 yrs)	Post-traumatic OA (66%); Primary OA (26%); RA (8%)	Group (1): HINTEGRA (40%); Group (2): MOBILITY (60%); 31 mo	Group (1): Ankle impingement (38%) *; Osteolysis (19%); Neuralgia (14%); Revision (10%). Group (2): Osteolysis (12%); Neuralgia (15%); Revision (9%); Ankle impingement (9%)	Group (1): 33%; Group (2): 15%.
Jung, 2016 [37]	Retrospective comparative case series (2004–2012)	52/54 (24/28; Mean 65 yrs)	Post-traumatic OA (67%); Primary OA (26%); RA (6%); Post-infection sequelae (1%)	Group (1): HINTEGRA (39%); Group (2): MOBILITY (61%). 31 mo	n.r.	Group (1): total (33%). Posterior (86%), Anterior (14%). Group (2): total (15%). Anterior (83%), Anterior–posterior (17%)
Rushing, 2022 [38]	Retrospective comparative case series (2015–2018)	90/90 (48/42; Mean 60 yrs)	Post-traumatic OA (71%); Primary OA (18%); Inflammatory arthritis (3%); Recurrent instability (6%)	Group (1): INFINITY (69%); Group (2): CADENCE (31%). 24 mo	Re-operation (2%)	Group (1): total (56%). Anterior (19%), Posterior (23%), Anterior–Posterior (15%). Grade I (37%), Grade II (40%), Grade III (17%), Grade IV (6%). Group (2): total (54%). Anterior (14%), Posterior (39%). Grade I (40%), Grade II (40%), Grade III (20%)
Doyle, 2022 [39]	Retrospective comparative case series (2017–2020)	71/71 (n.r.)	n.r.	Group (1): INFINITY (57%); Group (2): CADENCE (23%); Group 3): VANTAGE (20%). 20 mo	n.r.	Group (1): total (81%) *. Grade I (58%), Grade II (15%), Grade III (24%), Grade IV (3%). Group (2): total (50%). Grade I (50%), Grade II (25%), Grade III (25%). Group 3): total (57%). Grade I (100%)
Nunley, 2018 [40]	Prospective randomized (2011–2014)	84/84 (n.r.; Mean 65 yrs)	n.r.	Group (1): STAR (49%); Group (2): Salto Talaris (51%). 54 mo	Group (1): Lucency/cyst (51%), Subsidence (29%), Re-operation (20%); Group (2): Lucency/cyst (23%), Subsidence (2%), Re-operation (7%)	Group (1): total (61%) **. Group (2): total (30%)
Togher, 2023 [41]	Retrospective comparative case series (2016–2021)	22/22 (14/8; Mean 64 yrs)	n.r.	Group (1): Smooth-stemmed INBONE II (50%); Group (2): Fully porous-coated stemmed INBONE II (50%). 28 mo	Group (1): Revision (45%); Re-operation (9%). Group (2): Periprosthetic tibial cyst (55%); Re-operation (9%)	Group (1): total (55%). Group (2): total (46%)

* *p* < 0.05: Group (1) Vs. Group (2); **, *p* < 0.005: Group (1) Vs. Group (2).

### 3.1. Study Characteristics

**Figure 2:**
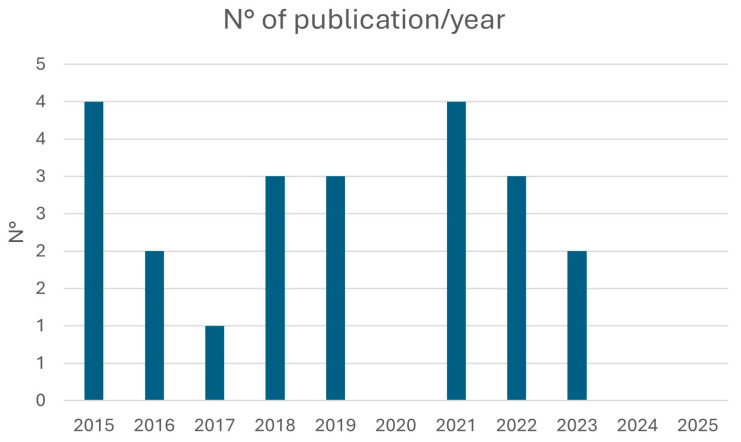
Histogram on the number of publications per year from February 2015 to February 2025.

Publication years ranged from 2015 to 2025, with peak output in 2015 and 2021 (4 studies each). No eligible articles were published in 2020, 2024 or 2025 (Figure 2).

**Figure 3:**
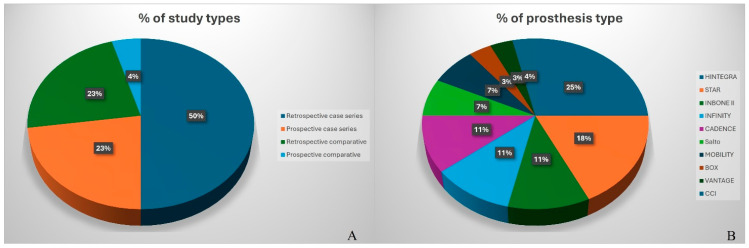
Pie charts: (**A**) percentage (%) of study types of the included articles; (**B**) percentage (%) of prosthesis employed in the included articles.

Among the 22 studies, 16 were case series [7,12,22,23,24,25,26,27,28,29,30,31,32,33,34,35], with 11 being retrospective [12,22,23,25,26,27,29,30,31,32,33] and 5 prospective [7,24,28,34,35], while 6 were comparative studies [36,37,38,39,40,41], with 5 being retrospective [36,37,38,39,41] and 1 prospective randomized [40] (Figure 3A).

### 3.2. Surgical Indications

Post-traumatic and primary osteoarthritis were the predominant indications for TAA (>90%), followed by rheumatoid arthritis, especially in studies involving the STAR prosthesis. Less common etiologies included AVN, inflammatory arthritis, and revision cases. Several studies did not specify the underlying diagnosis [27,30,35,39,40,41].

HO presence was reported in all studies, with severity grading available in 11/22 articles using the Brooker classification modified by Lee [12,22,25,27,28,29,30,31,32,38,39] (Table 2).

### 3.3. Surgical Approach

All prostheses evaluated were implanted through an anterior approach, precluding comparative analysis of surgical access on HO development. No approach-specific risk factors were identified.

### 3.4. HO and Coronal Alignment or ROM

Only one study [12] addressed coronal alignment, reporting no significant correlation with HO. However, a weak link between varus malalignment and anterior/lateral HO was noted. ROM analysis, incorporating data from four studies [7,22,24,32], showed no statistically significant relationship between ROM change and HO incidence (*p* = 0.958) (Table 3).

### 3.5. Implant Design

#### 3.5.1. Case Series by Implant Design

Among the 22 included studies, 13 case series evaluated specific implant types. The most frequently assessed prostheses were HINTEGRA (5 studies), STAR (4), INBONE II (2), INFINITY (1), and CADENCE (1). Additional devices included BOX, Salto Mobile-Bearing, CCI, and VANTAGE (Figure 3B).

HINTEGRA was examined in five studies [7,12,22,23,24], including 471 patients (M:F ratio 1:0.68; age range: 55–69 years). HO incidence varied markedly: 1% [23], 48% [22], and 99% [12], with follow-ups of 45, 36, and 77 months, respectively. Lee et al. reported a lower incidence (~7%) in prospective cohorts (*n* = 144 and 123) after longer follow-ups (88 and 78 months). HO was mostly posterior [12,22], and Grade I was the most frequent severity [22]. Major complications included osteolysis (up to 48%), cysts (40%), and reoperation (13–28%).

STAR was analyzed in four studies [25,26,27,28] with 252 patients (M:F ratio 1:1.53; age range: 59–74 years). HO incidence was consistently high: 100% [25], 78% [26], 62% [27], and 98% [28], predominantly at Grade III. The most frequent complications were lucency (56%), swelling (55%), and osteolysis (14%). Talar subsidence (6–9%) and reoperation rates (5–9%) were reported.

INBONE II was studied in two retrospective series [29,30] (*n* = 43; M:F ratio 1:0.72; mean age: 63–68 years). HO incidence ranged from 31% [29] to 67% [30], mostly posterior and of Grades I and III. Complications included component subsidence (31%), reoperation (33%), osteolysis (17%), and isolated peroneal nerve injuries (3%).

BOX (Bianchi et al. [31], *n* = 34) showed a 91% HO rate (mostly Grades III–IV) at 143-month follow-up, with a lucency rate of 94% and cysts in 6%.

Salto Mobile-Bearing (Wan et al. [32], *n* = 59) was associated with 22% HO (Grades I–II), 46% osteolysis, and a 12% reoperation rate at 36 months.

CCI (Van Es et al. [33], *n* = 237) showed 13% HO after 83 months, with 22% revision and 53% reoperation rates.

INFINITY (Penner et al. [34], *n* = 67) demonstrated a low HO rate (3%) with minimal complications (3% revision, 9% reoperation) at 35 months.

CADENCE (Rushing et al. [35], *n* = 32) had no HO reported after 24 months; the most common complication was lucency (41%), with a 31% reoperation rate.

Although comparisons are limited by heterogeneity in follow-up and methodology, INFINITY was associated with the lowest reported HO incidence, predominantly anterior when present. However, no consistent statistical significance was observed across designs.

#### 3.5.2. Comparative Studies

HINTEGRA vs. MOBILITY [36,37]: HO incidence was higher in HINTEGRA (33%) than MOBILITY (15%), with posterior vs. anterior localization, respectively. Differences were not statistically significant.

INFINITY, CADENCE, VANTAGE [38,39]: HO incidence ranged from 54% to 57%, with INFINITY showing significantly higher rates than CADENCE (*p* = 0.04).

STAR vs. SALTO [40]: STAR had a significantly higher HO rate (61% vs. 30%; *p* < 0.01).

INBONE II (stem types) [41]: No significant difference in HO between porous-coated (55%) and smooth-stemmed (46%) implants.

These studies suggest trends in implant-specific HO localization and rates, though significance was inconsistent.

### 3.6. HO Incidence and Radiographic Follow-Up

No significant association was found between HO incidence and radiographic follow-up duration (*p* > 0.05). The Durbin–Watson test confirmed the absence of autocorrelation (*p* = 0.292), and the regression intercept was not significant (*p* = 0.488).

### 3.7. HO and Reoperation

Approximately 6.5% of TAAs underwent surgical excision of HO. Follow-up duration was significantly correlated with resection rate (*p* = 0.009), though unrelated to total reoperation frequency (*p* = 0.179).

Multiple linear regression models analyzing Brooker Grades I–III did not yield statistically significant associations with the following resection rates:

Grade I and II model: R^2^ = 0.419; *p* > 0.05.

Grade II and III model: R^2^ = 0.0517; *p* > 0.05.

Grade IV was excluded due to limited data. Overall reoperation rate showed no correlation with HO incidence, resection rate, infection, subsidence, or loosening. The model’s intercept (0.186) was consistent with a mean reoperation rate of 22.2%, indicating multifactorial causes.

### 3.8. Risk of Bias

The Downs and Black scores averaged 13.4 (range: 9–18), indicating low overall study quality. The mean modified Coleman methodology score was 38 (range: 24–60), reinforcing the limited methodological robustness across the included studies (Table 4).

## 4. Discussion

The growing success of TAA, supported by the development of fourth-generation implants and a progressive increase in annual procedural volumes in both European and North American registries [42,43,44] potentially leading to early osteoarthritis in adjacent joints, TAA aims to restore physiological joint mobility, relieve pain, and improve overall patient quality of life.

Despite these advantages, TAA has shown complication and failure rates comparable to those of arthrodesis [45] such as HO, osteolysis, cyst formation, and peri-implant radiolucency frequently reported among mid- to long-term complications [8,46].

HO, a well-recognized complication in other joints such as the elbow and, most notably, the hip in prosthetic surgery [47,48,49], has also been documented following TAA. However, despite its recurrent mention in the literature, reported incidence rates vary widely depending on the type of prosthesis—and even within the same design—reflecting a lack of consensus [10].

A contributing factor to the limited understanding of this complication is the scarce attention it receives in clinical studies: many authors do not report HO as a specific outcome following TAA, hindering efforts to determine its actual incidence and prevalence across patient populations and implant types. Consequently, orthopedic surgeons are often unable to derive optimal intraoperative or postoperative prevention strategies. Moreover, it remains unclear whether HO represents a true complication or rather a frequent, benign postoperative finding with minimal clinical implications.

In this review, only 22 clinical studies were identified over a 10-year span, with most showing low methodological quality: just one randomized controlled trial (RCT) was found [40], while the vast majority were retrospective (17/22) [7,16,22,23,25,26,27,29,30,31,32,33,36,37,38,39,41], and only six were comparative in nature [36,37,38,39,40,41]. Two independent risk-of-bias assessments confirmed the generally poor quality of the evidence.

Common methodological limitations included the absence of power analysis, randomization, and blinding, as well as insufficient control of confounding factors. Furthermore, more than half the studies (55%) reported concomitant procedures, while surgical (68%) and rehabilitation (59%) protocols were often poorly described. None of the studies included MRI or histological evaluations.

Nevertheless, follow-up duration and sample size were acceptable in most studies: 57% enrolled more than 60 patients, and 95% reported a follow-up between 24 and 60 months. Clinical outcome measures, assessment methods, and inclusion criteria were generally well detailed. Follow-up ranged from 2 years [35,38,39,41] to 15 years [27], with 3-year [17,22,29,32,34,36,37] and 6–7-year follow-ups [7,23,24,28,30,33] being most common.

Except for two studies with fewer than 20 patients [26,30], sample sizes ranged from 22 [41] to 237 [33]. The mean patient age was 62 years, with a nearly equal female-to-male ratio (F/M 0.89), and most procedures were unilateral. The predominant indication for TAA was post-traumatic osteoarthritis, consistently reported across studies. TAA is often indicated in subjects over 65 years of age with advanced ankle arthritis, a common condition in the elderly, especially if they have had previous trauma or suffer from degenerative diseases. A recent study found that the median age of patients undergoing total ankle arthroplasty is 63 years, and this figure has remained constant over the past two decades [50].

Surgical indications were homogeneous, with post-traumatic ankle osteoarthritis being the most common indication for TAA. All procedures used the anterior approach, reflecting its widespread adoption. While this uniformity limits comparative analysis of surgical access impact on HO formation, it also underscores the approach’s advantages in joint exposure and deformity correction in the coronal plane. Nonetheless, a limitation of the anterior approach is its reduced control over sagittal alignment, which could affect implant biomechanics.

Although this review, supported by a recent systematic review [51], did not identify a clear correlation between HO and postoperative ROM, the potential association between HO and joint alignment, particularly in the coronal plane, remains insufficiently investigated. To date, only one study [16] has suggested a possible link between coronal alignment and HO, but the results did not reach statistical significance. Moreover, most available studies did not stratify alignment according to HO status, which limits the possibility of drawing definitive conclusions.

Prior investigations into alignment and postoperative ROM [52,53] did not find significant coronal plane correlations, but they did highlight the importance of sagittal alignment—particularly anterior translation and inclination of the talar component relative to the tibial component—in achieving better ROM. These findings suggest that sagittal parameters may play a more influential role in joint function after TAA.

Given the possible biomechanical contribution to HO, it is plausible that suboptimal sagittal alignment—harder to manage via the anterior approach—may facilitate ectopic bone formation through altered kinematics or loading. This points to the need for more focused studies on the relationship between surgical approach, multiplanar alignment, and the periarticular biological response.

In parallel, implant design may also influence HO incidence and anatomical distribution. Comparative and series data suggest that some prostheses predispose to distinct HO patterns. For example, the MOBILITY prosthesis demonstrated a lower HO rate (15%) than HINTEGRA (33%) in a matched comparison [37], but with different distribution—mainly anterior for MOBILITY and posterior for HINTEGRA. This anterior pattern may stem from deeper anterior tibial cuts needed for MOBILITY’s tibial component, possibly causing localized periosteal irritation and subsequent HO. HINTEGRA, offering broader anterior coverage, may mitigate this but redirect ossification posteriorly [16,22,23].

Similarly, INFINITY and CADENCE—despite shared lineage—had different HO rates in a direct comparison [39]: INFINITY showed 57% HO versus 54% in CADENCE, predominantly posterior in both. While the difference is small, it suggests that minor geometric features—such as anterior edge congruence or talar orientation—might affect stress distributions and thereby HO formation. Notably, CADENCE was the only implant to report zero HO cases in a separate series [35], though shorter follow-up must be acknowledged.

Prostheses like STAR and BOX exhibited high HO rates (62–100% and 91%, respectively) [25,26,27,28,31] and more severe grades (III–IV), which may reflect design limitations, longer follow-up, or surgical/postoperative protocol differences. In contrast, INFINITY [34], Salto [32], and CCI [33] had lower HO rates (3–22%) and less severe grades, potentially linked to conservative bone resection or optimized bone-implant interfaces.

These findings support the hypothesis that implant-related factors—such as anterior tibial resection extent, cortical coverage, and component geometry—may influence HO formation patterns following TAA. For example, in comparative studies, the CADENCE prosthesis, requiring aggressive anterior tibial osteotomy, showed a predilection for anterior HO, whereas the HINTEGRA model, with anterior cortical reference and broad anterior coverage but less posterior support, was associated with predominantly posterior HO. However, due to heterogeneity in study design and reporting, these associations remain speculative and require further biomechanical and prospective investigation.

No significant relationship was observed between follow-up duration and HO incidence, suggesting that the underreporting of HO is likely due to inconsistencies in follow-up imaging protocols rather than temporal factors alone.

Although HO is a frequent radiographic finding after TAA, our analysis indicates that it does not consistently lead to revision. Only a minority of patients underwent surgery specifically for HO removal, and no linear association was found between HO severity (Brooker grade) and reoperation. This suggests that HO alone is rarely a direct revision indication. Instead, reintervention appears more likely when HO coexists with complications such as impingement, subsidence, osteolysis, or infection. The absence of a significant link between total reoperation rate and HO-related variables, along with a stronger correlation between follow-up duration and HO excision, further supports that while symptomatic HO may warrant surgery, most cases remain asymptomatic and are managed conservatively. These findings align with studies indicating that high-grade HO may contribute to pain and ROM limitation, but its clinical impact varies and should be interpreted in context. Therefore, surgical decisions should consider the overall biomechanical and functional status rather than HO presence alone.

Despite the frequent radiographic detection of HO following TAA, its diagnosis and prognostic value remain limited by several factors. Currently the Brooker classification modified for the ankle, are inconsistently applied across studies, impeding direct comparisons and clinical extrapolation. Moreover, the lack of routine postoperative imaging protocols and variability in follow-up duration contribute to underreporting and inconsistent detection of HO.

From a diagnostic standpoint, no specific biochemical markers or imaging modalities beyond standard radiographs have demonstrated consistent utility in predicting or identifying clinically relevant HO. While exploratory studies have assessed the role of markers of bone metabolism or inflammatory mediators, none have been validated for clinical use in TAA settings [54]. The prognostic significance of HO is similarly unclear. This review found no consistent correlation between HO severity and postoperative ROM, pain, or reoperation rates. Notably, even high-grade ossifications were frequently asymptomatic and did not necessitate revision surgery. When reoperation occurred, it was typically due to mechanical complications such as impingement or osteolysis, rather than HO severity alone.

Considering this, there is a pressing need for high-quality prospective studies incorporating standardized diagnostic criteria, longitudinal imaging, and functional outcome measures. Such efforts would enable the development of validated prognostic models, helping clinicians identify which patients are at genuine risk of HO-related morbidity and may benefit from targeted prophylactic or surgical interventions.

Based on the findings of this systematic review, several practical considerations can be drawn for clinical practice. First, HO after TAA is frequent but often clinically silent; surgical excision should be reserved for symptomatic cases causing impingement or functional limitation. Second, while implant design may influence the anatomical distribution of HO, current evidence does not support selecting a specific prosthesis solely to reduce its incidence. Third, meticulous surgical technique—minimizing soft tissue trauma, ensuring adequate irrigation, and removing bone debris—may help reduce the risk of HO formation in high-risk patients. Finally, standardized radiographic follow-up and early detection are recommended to identify cases that may benefit from closer monitoring or intervention, although no validated biochemical or imaging biomarkers currently exist to guide prophylaxis or early treatment.

## 5. Conclusions

HO is a frequently reported radiographic finding following TAA, with incidence and distribution patterns varying across implant designs. However, current evidence—largely retrospective and methodologically limited—fails to demonstrate a consistent association between HO severity and adverse clinical outcomes such as reduced ROM, pain, or revision surgery. Most cases appear asymptomatic and are managed conservatively.

Given the variability in HO reporting, absence of standardized diagnostic criteria, and limited prognostic value, HO should be interpreted as a common postoperative radiographic phenomenon with unclear clinical relevance. While some implant designs may influence its distribution, no definitive risk factors have been identified.

## Figures and Tables

**Table 2:** Brooker classification modified by Choi and Lee.

Class	Criteria
0	No heterotopic ossification
I	Islands of bone within the soft tissue about the ankle
II	Bone spurs from the tibia or talus, reducing the posterior joint space by <50%
III	Bone spurs from the tibia or talus, reducing the posterior joint space by ≥50%
IV	Bridging bone continuous between the tibia and the talus

**Table 3:** Association between HO and coronal alignment or ROM.

Study	Alignment	Post-op ROM	Comment
Lee, 2018 [7]	Varus: dorsiflexion 9° → 10°, plantarflexion 22° → 26° Valgus: dorsiflexion 9° → 11°, plantarflexion 20° → 25° Neutral: dorsiflexion 8° → 10°, plantarflexion 20° → 27°	ROM improvements observed across all groups, with no statistically significant differences	Coronal plane alignment does not appear to significantly influence ROM
Clifton, 2021 [23]	Valgus: ROM 17° pre-op → 8° post-opVarus: ROM 11° pre-op → 6° post-op	Decreased ROM postoperatively, but more neutral component positioning	Slight trend toward improved outcomes with more neutral alignment; weak correlation with ROM
Wan, 2018 [32]	Not specified	ROM improved (from 33° to 40°)	No association found between ROM and alignment
Bianchi, 2021 [31]	α, β, γ angles remained stable over time	ROM remained nearly unchanged	No significant correlation with implant orientation
Jung, 2015/2016 [36,37]	Some implants in varus; MOBILITY group in neutral alignment	ROM improvements noted particularly in MOBILITY-neutral group	Tendency toward better ROM with neutral alignment
Manegold, 2017 [12]	Varus alignment (>92°)	Not directly assessed for ROM	Weak correlation between varus alignment and heterotopic ossification (HO) in anterior/lateral recesses; excessive HO may contribute to joint stiffness

**Table 4:** Risk of bias.

Downs and Black Checklist							Modified Coleman Methodology Score		
Article	Reporting	External Validity	Internal Validity Bias	Internal Validity Confounding	Power	Total Score	Part A	Part B	Total Score
Deleu, 2015 [22]	8	3	3	2	0	16	40	30	10
Manegold, 2017 [12]	6	2	3	2	0	13	35	25	10
Clifton, 2021 [23]	6	2	2	3	0	13	40	30	10
Lee, 2018 [7]	9	3	4	3	0	19	54	44	10
Lee, 2019 [24]	8	3	3	2	0	16	54	44	10
Haytmanek, 2015 [25]	8	3	3	3	1	18	37	27	10
Jastifer, 2015 [26]	6	3	3	3	0	15	24	14	10
Palanca, 2018 [27]	6	3	3	3	0	15	28	18	10
Kerkhoff, 2016 [28]	9	3	3	3	0	18	60	50	10
Jamjoom, 2022 [29]	7	3	2	2	1	15	28	18	10
Rushing, 2021 [30]	8	2	3	2	0	15	29	19	10
Bianchi, 2021 [31]	7	3	3	3	0	16	28	18	10
Wan, 2018 [32]	8	2	3	3	0	16	29	19	10
Van Es, 2022 [33]	9	3	3	3	0	18	39	29	10
Penner, 2019 [34]	9	3	3	3	0	18	44	34	10
Rushing, 2021 [35]	7	2	3	2	0	14	38	28	10
Jung, 2015 [36]	7	3	3	2	0	15	42	32	10
Jung, 2016 [37]	8	3	3	3	0	17	44	34	10
Rushing, 2022 [38]	7	2	3	2	0	14	32	22	10
Doyle, 2023 [39]	5	3	2	2	0	12	50	40	10
Nunley, 2019 [40]	7	3	3	5	0	18	29	19	10
Togher, 2023 [41]	7	2	2	3	1	15	40	30	10

## Data Availability

The original contributions presented in the study are included in the article material, further inquiries can be directed to the corresponding author.

## Supplementary Materials

The following supporting information can be downloaded at: https://www.mdpi.com/article/10.3390/diagnostics15172203/s1, Figure S1: Schematic representation summarizing the PICOS elements applied in the systematic review.

## Abbreviations

The following abbreviations are used in this manuscript:

HO	Heterotopic ossification
TAA	Total ankle arthroplasty
NSAIDs	Nonsteroidal anti-inflammatory drugs
ROM	Range of motion
mCMS	Modified Coleman Methodology Score
AVN	Avascular necrosis
CCI	Ceramic coated implant
F	Females
f-up	Follow-up
i.o.	Intra-operative
M	Males
mo	Months
n.r.	Not reported
OA	Osteoarthritis
PE	Polyethylene
Pts	Patients
RA	Rheumatoid arthritis
Ref.	Reference
yrs	Years
