# Supplementary material for: Diagnostic Imaging and Clinical Implications of Heterotopic Ossification After Total Ankle Arthroplasty: A Systematic Review for Surgical Strategy
Source: Diagnostics (Basel). 2025 Aug 29;15(17):2203. doi: 10.3390/diagnostics15172203 (PMC12427652; doi:10.3390/diagnostics15172203)

Figure S1. Schematic representation summarizing the PICOS elements applied in the systematic review

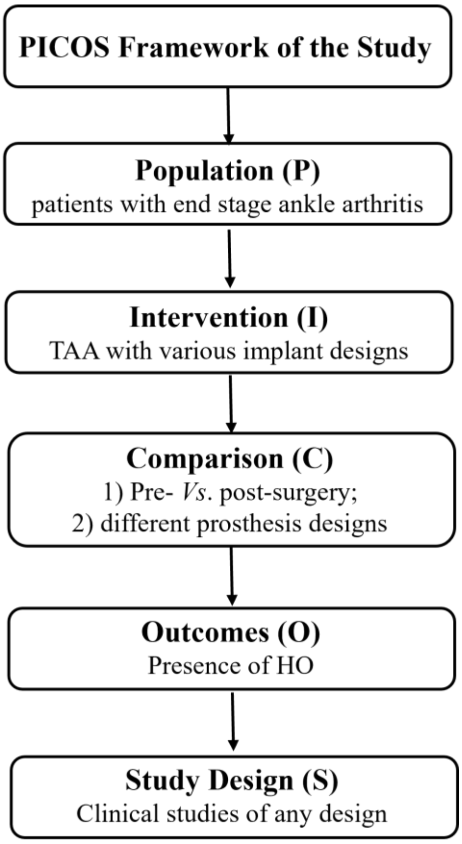

Supplement: Supplementary file 1 [file diagnostics-15-02203-s001.zip › diagnostics-3782159-supplementary.pdf]
